# Case Report: Diagnostic odyssey in rare diseases: when genetic variants are misinterpreted

**DOI:** 10.3389/fped.2026.1803924

**Published:** 2026-04-15

**Authors:** Minerva Montero-Hernández, Nahuel Pérez-Moix, María-Carmen Carrascosa-Romero, María-Pilar López-Garrido, Francisco Sánchez-Sánchez

**Affiliations:** 1 Medical Genetics Laboratory. Albacete School of Medicine, University of Castilla-La Mancha (UCLM), Albacete, Spain; 2 Biomedicine Institute, University of Castilla-La Mancha (UCLM), Albacete, Spain; 3 Neuropediatrics Service, Complejo Hospitalario Universitario de Albacete, Albacete, Spain; 4 Medical Genetics Laboratory, Ciudad Real School of Medicine University of Castilla-La Mancha (UCLM), Ciudad Real, Spain

**Keywords:** diagnostic odyssey, Marfan syndrome, misdiagnosis, *MYH11*, precision medicine, rare diseases, variant interpretation

## Abstract

**Introduction:**

The diagnostic odyssey in rare diseases often involves the misinterpretation of genetic data, particularly when multidisciplinary approaches are lacking. This study illustrates the critical process of interpreting variants from next-generation sequencing (NGS) through a real-life case of a child misdiagnosed with Marfan Syndrome (MFS). The misdiagnosis was maintained for over seven years despite repeated clinical evaluations by different specialists. An initial clinical suspicion of MFS due to joint hypermobility at 3 years of age became a definitive diagnosis after an external laboratory reported a heterozygous variant in the *MYH11* gene at age 5, despite the patient never fulfilling the established clinical diagnostic criteria for the disease.

**Methods:**

To provide an accurate diagnosis and end the family's diagnostic odyssey, a complete clinical and genetic reinterpretation was performed when the patient was 7 years old. The proband and 9 asymptomatic relatives were recruited for a functional study of the *MYH11* c.5544_5548del, p.(D1848Efs*60) variant.

**Results:**

The functional analysis demonstrated that the variant operates through a loss-of-function mechanism, leading to nonsense-mediated mRNA decay. While gain-of-function variants in *MYH11* are associated with thoracic aortic aneurysms and dissections, loss-of-function variants are linked to autosomal recessive Megacystis-Microcolon-Intestinal Hypoperistalsis Syndrome (MMIHS). As a heterozygous carrier of a loss-of-function variant, the patient is asymptomatic for MMIHS and definitively does not have MFS. Currently, the 11-year-old child is progressing favorably without any notable pathology.

**Conclusions:**

This case exposes the entire diagnostic odyssey suffered by the patient's family and highlights three fundamental systemic errors: the critical delay in genetic counseling, the over-interpretation of NGS data by external laboratories lacking phenotypic context, and the health system's inefficiency in integrating clinical geneticists. Overcoming these barriers is essential for the true implementation of personalized precision medicine.

## 1 Introduction

The term “Diagnostic Odyssey” is increasingly used in the field of rare diseases to designate the prolonged period that elapses from the moment a symptom or abnormal feature is first noted until a definitive diagnosis is made. Although rare diseases can arise at any age, they are predominant in the pediatric population, meaning this odyssey profoundly affects both the patients and their parents. When the time to diagnosis exceeds one year, it is considered a diagnostic delay, but the average of this period can be protracted, in rare cases, to 5–8 years (1–6). It is, therefore, a slow, stressful, and costly process, characterized by clinical uncertainty, emotional distress, numerous tests, and recurrent hospitalizations, during which parents struggle to find answers without success.

The integration of Next-Generation Sequencing (NGS) into clinical practice over a decade ago represented a breakthrough in the diagnosis of rare diseases, albeit with uneven implementation depending on national legislation and healthcare resources. The identification of novel disease-causing genes has considerably shortened diagnostic timelines and increased our understanding of the metabolic pathways involved, leading to the design of precision drugs for new therapeutic targets. However, the diagnostic odyssey is not over for many of these patients (7–10). The integration of an individual's genetic and genomic data with their clinical and exposome profiles forms the basis of personalized precision medicine, an emerging field that addresses disease prevention, diagnosis, and treatment, representing a new paradigm in healthcare systems (11, 12). Full implementation of this concept in routine clinical practice has not yet been achieved. In the meantime, hospitals urgently need to incorporate professionals with genomics competencies to perform adequate genetic diagnoses and provide appropriate counseling to patients.

Marfan syndrome (MFS; OMIM #154700) is a rare but well-defined disease in terms of etiology, symptomatology, and diagnostic criteria (Table 1; Supplementary Table S1). It affects approximately 1 in 5,000 to 1 in 10,000 live births (13, 14) and is associated with a reduced life expectancy if an accurate diagnosis and proper follow-up are not provided. MFS belongs to a heterogeneous group of connective tissue pathologies characterized by collagen and/or extracellular matrix defects. These conditions include Shprintzen-Goldberg syndrome, Loeys-Dietz syndrome, vascular Ehlers-Danlos syndrome, Weill-Marchesani syndrome, and Beals-Hecht syndrome, which are caused by pathogenic variants in different genes but share overlapping clinical features. Given this phenotypic heterogeneity and symptom overlap, achieving an accurate genetic diagnosis is essential. To achieve a faster differential diagnosis, recent strategies suggest the use of clinical exome sequencing followed by the analysis of phenotype-driven virtual gene panels, which include genes like *FBN1* (the primary cause of MFS), thereby optimizing resources and reducing the diagnostic odyssey (15).

**Table 1:** Marfan syndrome diagnosis criteria according to the 2010 revised Ghent nosology (16).

Family history	Clinical features
In absence of family history	1*. Dilatation or dissection of the aortic root (z-score≥2). Ectopia lentis.
	2. Dilatation or dissection of the aortic root (z-score≥2). No ectopia lentis but identification of a pathogenic variant in the *FBN1* gene.
	3*. Dilatation or dissection of the aortic root (z-score≥2). No ectopia lentis or mutations in *FBN1* but other systemic findings (scoring system ≥*7*; Supplementary Table S1)
	4. Ectopia lentis. No aortic involvement but known pathogenic variant in *FBN1* gene.
With family history	5. Ectopia lentis.
	6*. Systemic findings (scoring system ≥7; Supplementary Table S1)
	7*. Dilatation or dissection of the aortic root (*z*-score ≥2 before the age of 20; *z*-score ≥3 after the age of 20).

Z-score: number of standard deviations away from the mean for age and body surface area. A Z-score ≥2 implies a percentile greater than or equal to 95, whereas one ≥3 implies a percentile greater than or equal to 99. *: in the absence of differential diagnosis of Shprintzen-Goldberg syndrome, Loeys-Dietz syndrome or vascular Ehlers-Danlos syndrome.

Another non-syndromic disorder related to this group of pathologies is thoracic aortic aneurysm and aortic dissection (TAAD; OMIM #132900), which can present with or without patent ductus arteriosus. The latter is caused, among other factors, by heterozygous pathogenic variants in the *MYH11* gene (HGNC:7569) that exert a gain-of-function (GOF) or dominant-negative effect on the encoded protein, following an autosomal dominant inheritance pattern (17–20). This gene, located in the 16p13.13-p13.12 region, encodes the smooth muscle myosin heavy chain (myosin-11), the major component of the thick contractile filaments in smooth muscle cells (21).

Conversely, loss-of-function (LOF) variants in the *MYH11* gene, when present in homozygosity or compound heterozygosity, cause Megacystis-Microcolon-Intestinal Hypoperistalsis Syndrome (MMIHS; OMIM #619351), an autosomal recessive disorder (22–26). Therefore, deeply understanding the precise molecular implications (GOF vs. LOF) of each identified variant in this gene is critical to establish a correct genotype-phenotype association.

With the specific case described in the present study, we aim to expose the mistakes made in the clinical and genetic diagnosis of a child presenting with joint hypermobility. This case serves as a paradigm to draw attention to the risks of misinterpreting genetic variants when there is a lack of multidisciplinary communication and a failure to integrate clinical geneticists into the routine diagnostic workflow.

## 2 Subjects and methods

### 2.1 Participants in the study

A 7-year-old child, previously diagnosed with Marfan Syndrome, was recruited at the Neuropediatric Unit of the Complejo Hospitalario Universitario de Albacete, Spain. The aim was to complete a molecular genetic analysis and investigate the potential association of a previously identified *MYH11* variant (detected via NGS) with the proband's phenotype. At the time of recruitment, the child presented with joint hypermobility and difficulties in both fine and gross motor skills. Additionally, he was wearing a number 4 ankle-foot orthosis (AFO) due to femoral anteversion secondary to the hypermobility. Cardiological and ophthalmological evaluations revealed no pathological findings.

The child's parents were also recruited for the initial genetic study. Once the preliminary genetic results were obtained, the study was expanded to include his younger brother and the closest paternal relatives: the grandparents, an aunt, and two first cousins of the proband. All of them are asymptomatic for the clinical signs presented by the proband.

The proband's medical history was systematically collected through the Neuropediatrics Unit. The genetic analyses were performed within the scope of routine clinical diagnostic assistance rather than a prospective research trial. Specifically, this diagnostic work was conducted under an official institutional agreement for precision diagnostics between the regional public health service (Servicio de Salud de Castilla-La Mancha, SESCAM) and the University of Castilla-La Mancha (UCLM). Under this legal framework, individual clinical diagnostic cases do not require separate approval by the local Ethics Committee. However, all procedures were conducted in accordance with the Declaration of Helsinki. Written informed consent for the genetic testing and for the publication of this case report was obtained from the proband's parents and all adult participants.

### 2.2 Genetic analysis

#### 2.2.1 DNA extraction

Genomic DNA (gDNA) was extracted from peripheral blood leukocytes of all subjects using the E.Z.N.A. Blood DNA Kit (Omega Bio-tek), following the manufacturer's protocol. DNA concentration and purity were quantified using a NanoDrop ND-1000 spectrophotometer (Thermo Fisher Scientific).

#### 2.2.2 RNA extraction and cDNA synthesis

Total RNA was extracted from peripheral blood leukocytes of all subjects using the GeneJET RNA Purification Kit (Thermo Fisher Scientific) according to the manufacturer's instructions. RNA yield was quantified using the NanoDrop ND-1000 spectrophotometer.

Complementary DNA (cDNA) was synthesized from 1 μg of total RNA per sample using the SuperScript IV Reverse Transcriptase (Invitrogen, catalog no. 18090050) with a mixture of oligo(dT) and random hexamer primers, according to the manufacturer's protocol.

#### 2.2.3 Sanger sequencing

The identified variant was confirmed in the proband and analyzed in the remaining family members by Sanger sequencing. Specific PCR primers were designed to amplify the intronic regions flanking exon 39 of the *MYH11* gene: forward 5′-GGAAAGCACTCAAGATGCCACC-3′ and reverse 5′-TGCCTTTCTCTGCCTGTCGC-3′. PCR was performed in a 50 µL reaction volume containing 100 ng of gDNA, 10X standard reaction buffer with 2 mM MgCl₂, 10 mM dNTP mixture, 10 pmol of each forward and reverse primer, and 1 U of *Taq* DNA polymerase (Biotools). Thermocycling conditions consisted of an initial denaturation at 94°C for 5 min, followed by 40 cycles of 94 °C for 30 s, 63 °C for 30 s, and 72 °C for 30 s, with a final extension at 72 °C for 5 min.

Terminator cycle sequencing was carried out using the BigDye Terminator v3.1 Cycle Sequencing Kit (Applied Biosystems). The resulting sequencing reaction products were analyzed on a 3730xl automated DNA Analyzer (Applied Biosystems).

#### 2.2.4 Semi-Quantitative PCR and fragment analysis

Four microliters of total cDNA and 5 ng of gDNA were used for PCR amplification. The following primers were designed to target exons 39 and 40 of the *MYH11* gene: forward 5′-CCAGAGAGAAACAGGCGGC-3′ and reverse 5′-CTTGAGTGCGTTCACCTCG-3′. The expected cDNA PCR amplicon size was 270 bp, while the gDNA amplicon was 350 bp.

The PCR reaction was performed in a 30 µL volume containing 10X standard reaction buffer with 2 mM MgCl₂, 10 pmol of each primer, 10 mM dNTP mixture, and 1 U of *Taq* DNA polymerase (Biotools). Thermocycling consisted of an initial denaturation at 94 °C for 5 min, followed by 38 cycles of 94 °C for 30 s, 65 °C for 30 s, and 72 °C for 30 s, with a final extension at 72 °C for 5 min.

Semi-quantitative PCR was conducted by collecting a 2 µL aliquot between PCR cycles 20 and 32 to determine the cycle in which amplification of the variant allele first appeared, allowing for subsequent quantification.

Following amplification, 2 µL of the PCR product was mixed with 40 µL of formamide and 0.3 µL of GENESCAN® 400HD size standard (Applied Biosystems). Fragment analysis was performed on a 3730xl DNA Analyzer (Applied Biosystems), and Peak Scanner v1.0 software was used to visualize fragment lengths and quantify the corresponding peaks.

## 3 Results

**Figure 1:**
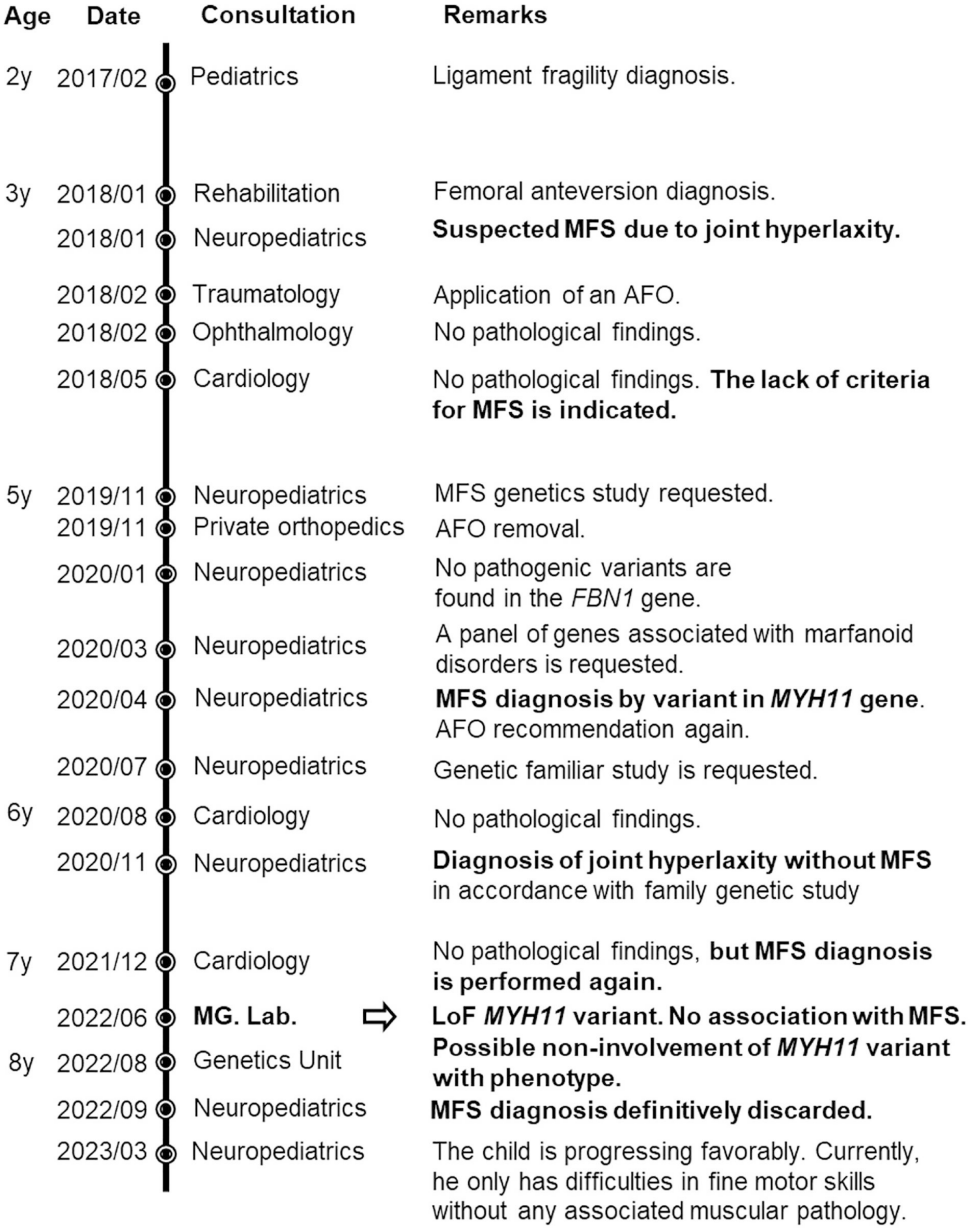
Timeline of the diagnostic odyssey suffered by the patient and his family for 7 years. The arrow indicates the moment at which it is determined that the *MYH11* variant is not associated with the patient's phenotype or with Marfan syndrome. MG lab, Medical Genetics laboratory; y, years.

The proband in this study was misdiagnosed with MFS based on the detection of a novel heterozygous variant in the *MYH11* gene [NC_000016.10(NM_022844.3):c.5544_5548del; NP_074035.1:p.(Asp1848Glufs*60)]. This misdiagnosis persisted for several years, fostered by an ambiguous NGS report from an external laboratory and a lack of multidisciplinary communication. Crucially, the patient never met the clinical diagnostic criteria for MFS (Table 1), nor did he present any symptoms associated with TAAD. The clinical timeline and genetic re-evaluation are detailed below in chronological order and summarized in Figure 1.

### 3.1 Clinical presentation and diagnostic odyssey

#### 3.1.1 2017 (2 years old)

The patient first attended the Pediatrics Service of the Spanish National Health System, where he was diagnosed with ligament flaccidity and referred to rehabilitation.

#### 3.1.2 2018 (3 years old)

During a follow-up evaluation, bilateral femoral anteversion was diagnosed, prompting a referral to the Neuropediatric Service. At this stage, a specialist raised the clinical suspicion of MFS for the first time due to the presence of joint hypermobility and a high-arched (ogival) palate. Traumatology specialists detected mild motor difficulties, confirmed the femoral anteversion, and prescribed a number 4 ankle-foot orthosis (AFO). Given the suspicion of MFS, the patient was referred to Ophthalmology and Cardiology. Both examinations were completely normal. The cardiology report explicitly indicated that the patient did not meet the formal criteria for MFS (aortic annulus diameter Z-score: −0.24; aortic sinotubular junction Z-score −0.49). Despite the lack of clinical evidence, no genetic testing was ordered, and the clinical suspicion of MFS was maintained.

#### 3.1.3 2019 (4 to 5 years old)

Motor clumsiness associated with joint hypermobility persisted, affecting both fine and gross motor skills. Due to the sustained clinical suspicion, a genetic test targeting the *FBN1* gene was requested, which returned negative results. Following this, NGS of a 10-gene panel associated with marfanoid phenotypes was outsourced to a private, external genetic company (details of the targeted gene panel are provided in the Supplementary Material).

#### 3.1.4 2020 (6 years old)

In March 2020, the external company issued a genetic report identifying a heterozygous variant in the *MYH11* gene (c.5544_5548del). The report explicitly concluded that this finding confirmed “*the Marfan syndrome and related disorders.”* The variant was absent from the general population (Genome Aggregation Database) and had not been previously associated with any phenotype in the literature. Bioinformatics predictors (MutationTaster, SIFT, and PolyPhen-2) estimated a pathogenic effect. The company's report mentioned the association of pathogenic *MYH11* variants with TAAD but erroneously used this to support the MFS diagnosis without considering the specific functional impact of the variant. According to the American College of Medical Genetics (ACMG) guidelines (27), a frameshift variant may carry strong evidence of pathogenicity; however, in this context, the interpretation was flawed because LOF variants in *MYH11* are not associated with TAAD.

In August 2020, a new cardiology check-up was performed due to the persistent MFS label. Once again, the results were completely normal (aortic annulus diameter Z-score: 0.88; aortic sinotubular junction Z-score −0.67). Shortly after, a different neuropediatrician questioned the MFS diagnosis, noting the lack of familial segregation for the phenotype, and suggested that the patient simply had benign joint hypermobility.

### 3.2 Segregation analysis and genetic Re-evaluation

**Figure 2:**
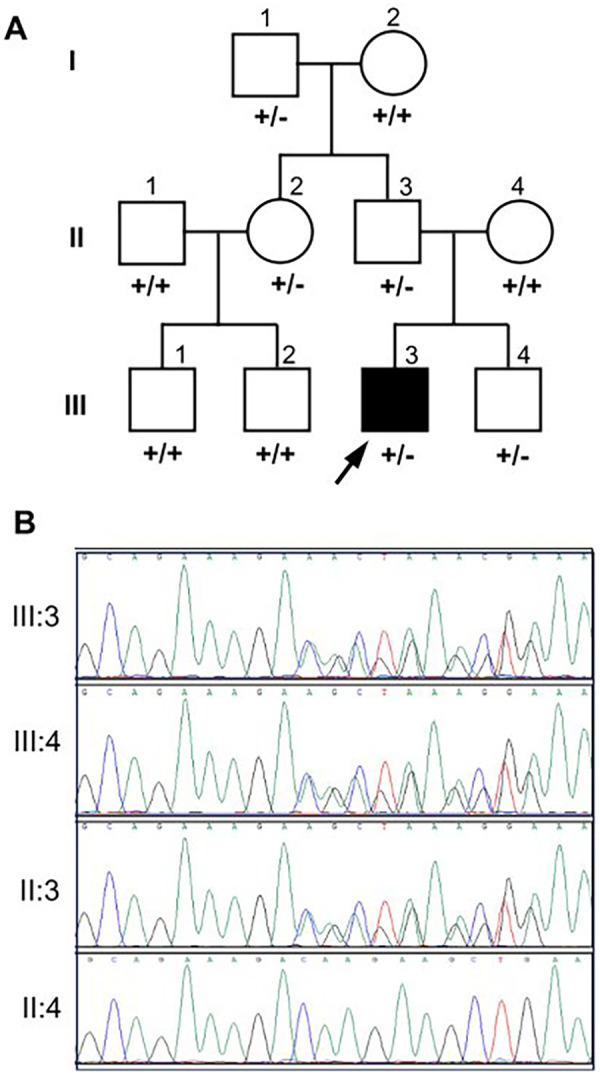
Genealogy of the family studied **(A)** and sanger sequences **(B)** of the proband (III:3), sibling (III:4), father (II:3) and mother (II:4) showing the deletion. +: wildtype allele; -: mutant allele [p.(Asp1848GlufsTer60) in the *MYH11* gene]. Arrow indicates the proband subject.

Following the genetic report, segregation analysis was recommended to determine if the variant was *de novo*. Weeks later, the variant was found to be inherited from the proband's completely asymptomatic father. The study was subsequently extended, identifying the same heterozygous variant in the paternal grandfather, a paternal aunt, and the proband's sibling (Figure 2A). Strikingly, all carrier relatives were completely asymptomatic. Comprehensive medical examinations, including echocardiograms, revealed no pathological findings in any of the carriers, not even in the 74-year-old grandfather. Despite this clear lack of genotype-phenotype segregation, the MFS diagnosis was not officially withdrawn for almost two more years.

Furthermore, the external genetic report completely failed to mention the established association between *MYH11* LOF variants and MMIHS. This distinction is critical, as all heterozygous individuals in this family are merely asymptomatic carriers of a recessive disease. Table 2 summarizes the distinct inheritance patterns and phenotypes associated with various *MYH11* variants, demonstrating that not all pathogenic variants follow an autosomal dominant inheritance pattern.

**Table 2:** Pathogenic variants found in the MYH11 gene associated to different phenotypes and inheritance patterns.

Genotype	State	Phenotype; Inheritance pattern	Reference
NP_002465.1:p.(R712Q)	Heterozygous	TAAD; AD	(18)
NP_001035202.1:p.(L1264P)	Heterozygous	TAAD; AD	(20)
NP_002465.1:p.(R1758Q)	Heterozygous	TAAD; AD	(17)
NP_001035202.1:p.(I1078M)	Heterozygous	TAAD; AD	(28)
NP_001035202.1:p.(P1940Hfs*91)	Heterozygous	CIPO/ SMDS; AD	(29, 30)
NP_001035202.1:p.(Q1941Nfs*91)	Heterozygous	SMDS; AD	(30)
NP_001035202.1:p.(R684H) NP_001035202.1:p.(E1180D,Q1181*)	Compound heterozygous	MMIHS; AR	(25)
16q13.11del (1.3Mb) NP_001035202.1:p.(P127S)	Compound heterozygous	MMIHS; AR	(22)
NP_002465.1:p.(K1200*)	Homozygous	MMIHS; AR	(24)
NP_001035202.1:p.(p.R937Gfs*7)			
NP_002465.1:p.(K1141Tfs*20)	Compound heterozygous	MMIHS; AR	(23)
NP_001035202.1:p.(R531*)	Homozygous	MMIHS; AR	(31)
NP_002465.1:p.(D1848Efs*60)	Heterozygous	Potencial MMIHS; AR	Present study

TAAD, thoracic aortic aneurysm and aortic dissections; CIPO, chronic intestinal pseudo-obstruction; SMDS, smooth muscle dysmotility syndrome; MMIHS, megacystic-microcolon-intestinal hypoperistalsis syndrome; AD, autosomal dominant; AR: autosomal recessive.

In June 2022, desperate for answers, the family arrived at our Medical Genetics research laboratory. We collected fresh blood samples and comprehensively reviewed the family's clinical history. We confirmed the presence of the variant via Sanger sequencing (Figure 2B). Upon review, we realized that the family had never been evaluated by the hospital's Clinical Genetics Service, and we immediately recommended a referral. Both our laboratory and the clinical genetics unit concluded that the *MYH11* variant was not responsible for the patient's phenotype.

Finally, in September 2022, at 8 years of age, the diagnosis of MFS was definitively withdrawn. Additional tests, including muscle enzyme analysis and electromyography, were negative, ruling out associated muscular pathologies. Currently, the 11-year-old child is progressing favorably; he no longer wears an AFO, plays sports, plays the clarinet, and exhibits only mild fine motor impairment without any associated muscular or cardiovascular pathology.

### 3.3 Functional analysis of the *MYH11* variant

To definitively confirm the pathogenicity mechanism of the variant, a functional evaluation was necessary. The c.5544_5548del variant is a five-nucleotide deletion located in exon 40 of the *MYH11* gene, predicting a frameshift and the introduction of a premature stop codon. We hypothesized that this would trigger the degradation of the mutant transcripts via the nonsense-mediated mRNA decay (NMD) pathway.

A semi-quantitative PCR assay was performed on total RNA extracted from the leukocytes of the proband, his sibling, and his parents. The synthesized cDNA was amplified and mixed with its corresponding genomic DNA (gDNA) amplified with the same primers for comparative purposes. The amplicon from the gDNA was longer than that from the cDNA because it contained intron 39, making it easily recognizable during fragment analysis.

**Figure 3:**
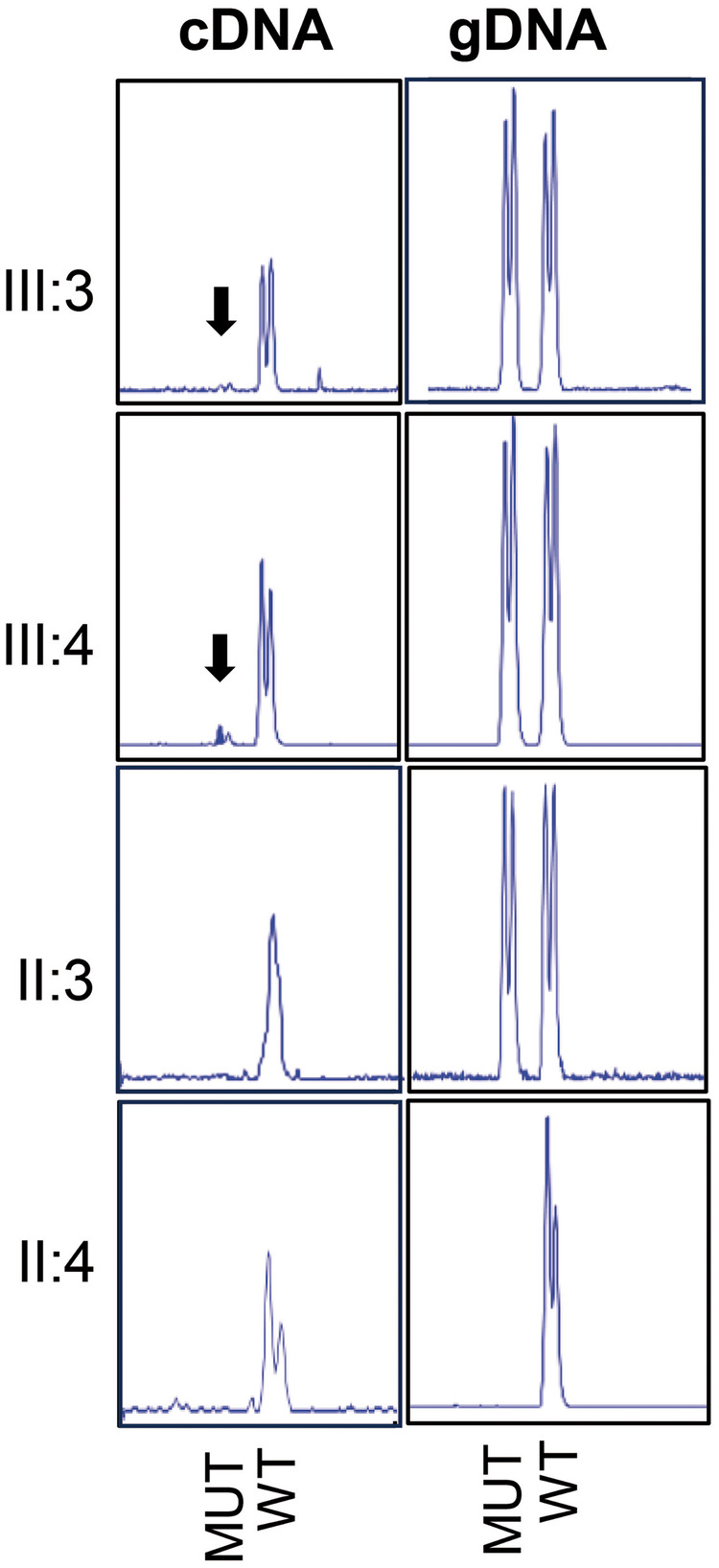
Fragment analysis of “exon39-exon40” of cDNA PCR (left panel) and “exon39-intron-exon40” of gDNA PCR (right panel) in the proband (III:3), sibling (III:4), father (II:3) and mother (II:4). cDNA PCR was stopped and quantified at cycle 30. The arrows show the residual mutant cDNA. MUT, mutant alelle; WT, wild type allele.

As shown in Figure 3, while the gDNA analysis confirmed the heterozygous status of the proband (III:3), sibling (III:4), and father (II:3), their respective cDNAs showed virtually no expression of the mutant allele compared to the wild-type allele. The mutant cDNA expression was unquantifiable in the proband, approximately 9% in the sibling, and 0% in the father. For the mother (II:4), her homozygous wild-type status was verified in both gDNA and cDNA.

These functional results conclusively demonstrate that the mutant transcripts are actively degraded by NMD. Therefore, the c.5544_5548del variant behaves as a null allele with a clear LOF effect, definitively ruling out any association with TAAD or MFS.

Figure 3 shows the heterozygous condition in the gDNA of the proband (III:3), sibling (III:4) and father (II:3), while in their respective cDNAs there is hardly any expression of the mutant allele in the proband compared to the wildtype allele (unquantifiable peak), around a 9% of expression in the sibling and 0% in the father. As for the mother (II:4), we verified her homozygous status in both gDNA and cDNA. In view of the results, we can show that the variant c.5544_5548del is null with a clear LOF.

## 4 Discussion

The proband of our study was misdiagnosed with MFS due to a heterozygous variant in the *MYH11* gene, even though he had no aortopathy, no *FBN1* pathogenic variant, nor any other symptoms meeting the diagnostic criteria for MFS. Through the case presented in this study, we expose the cascading mistakes made during the clinical and genetic diagnosis of a child with joint hypermobility. This diagnostic odyssey serves as a paradigm to draw attention to the misinterpretation of genetic variants, the lack of standardized protocols for suspected genetic diseases, and the poor communication among experts. Specifically, this case highlights the critical need for functional variant validation, the danger of over-interpreting genomic data without phenotypic context, the knowledge gap among non-geneticist physicians, and the severe consequences of delayed referral to genetic counseling.

### 4.1 Functional confirmation of the loss-of-function mechanism

Before addressing the systemic errors in the diagnostic process, it is essential to discuss the molecular findings that definitively resolved this case. To confirm the LOF nature of the c.5544_5548del variant, a semi-quantitative PCR assay was performed using RNA extracted from the proband and his family members. Since the variant is a five-nucleotide deletion, fragment analysis allowed us to detect and quantify both alleles separately. The results demonstrated a near-total absence of the mutant transcript in the heterozygous individuals, indicating that the nonsense-mediated mRNA decay (NMD) pathway actively prevents the synthesis of the truncated myosin-11 protein.

Interestingly, we observed slight inter-individual variability in NMD efficiency: while the father exhibited 0% mutant transcript, the proband showed an unquantifiable minimal residual percentage, and the sibling retained approximately 9%. It is well-documented that NMD efficiency can vary between individuals due to genetic background, developmental stage, or environmental factors (32–37). Furthermore, NMD performance can be tissue-specific (34, 38, 39), and our functional study was limited to blood-derived RNA. Nevertheless, the NMD system was sufficiently effective in all three individuals to trigger the degradation of the mutant transcripts.

Based on this functional evidence and following the ACMG guidelines (PVS1 criterion), we classify the c.5544_5548del variant as a null allele (LOF). This finding aligns with data reported by Kloth et al., who described three other *MYH11* frameshift variants (p.Lys1200Ter, p.Arg937Glyfs*7,* and p.Lys1141Thrfs20) that similarly induce mRNA degradation via NMD and are associated with MMIHS (22). Consequently, the proband and his heterozygous relatives are simply asymptomatic carriers of a recessive condition. This genotype is entirely unrelated to the patient's joint hypermobility, confirming that the *MYH11* variant was merely an incidental finding rather than the cause of a marfanoid phenotype.

### 4.2 The danger of over-interpretation by external laboratories

Having established the variant's true molecular mechanism, the initial misdiagnosis becomes even more striking. The external genetic company reported the novel variant and automatically linked it to MFS. However, pathogenic variants in this gene can cause two vastly different phenotypes depending on their mechanism of action. GOF or dominant-negative variants in heterozygosity cause TAAD (17–19, 40). These are predominantly missense or splice-site variants resulting in in-frame deletions (17). Conversely, LOF variants (such as the one functionally confirmed in our study) in homozygosity or compound heterozygosity cause MMIHS (22–26).

The external genomic facility attempted to interpret the NGS data without a clear vision of the patient's true clinical phenotype. By failing to assess the LOF nature of the frameshift variant, the laboratory incorrectly linked the finding to TAAD and erroneously confirmed an MFS diagnosis. When an external laboratory lacks comprehensive clinical information, it should strictly limit itself to issuing a genetic report based on standard ACMG classifications, avoiding any forced clinical interpretation that could mislead the prescribing physician.

### 4.3 Healthcare system inefficiencies and the genomics knowledge Gap

The misinterpretation of genetic variants identified by NGS is a matter of growing concern. Recent studies, such as the reanalysis of 152 rare disease exomes by Bartolomaeus et al., revealed that 18% of families received a clinically relevant change in diagnosis after five years (41). Diagnostic errors often stem from failing to confirm inheritance patterns or reporting pathogenic variants that do not logically explain the patient's phenotype (41–43).

Commercial genetic reports frequently include disclaimers delegating the ultimate responsibility for clinical interpretation to the prescribing physician. While this is methodologically appropriate, it exposes a critical inefficiency in the healthcare system: physicians across various specialties often possess limited knowledge of genetics. NGS reports are highly complex and laden with technical terminology, making them difficult to interpret accurately without specialized training. In our case, the physicians received a report diagnosing a marfanoid disorder and accepted it, despite the patient's phenotype clashing entirely with TAAD—which does not feature joint hypermobility as a primary sign (16)—and failing to meet the Ghent criteria. This underscores the risk of clinicians relying solely on laboratory conclusions without the specialized tools to critically evaluate the genotype-phenotype correlation.

### 4.4 Delayed genetic counseling and the cascade of errors

Perhaps the most significant error in this diagnostic odyssey was the extreme delay in referring the patient for genetic counseling. The patient was first evaluated by our Medical Genetics laboratory in June 2022, more than four years after the initial clinical suspicion of MFS (January 2018) and over two years after the erroneous NGS report. This wasted time translated into a profound health disservice, triggering a cascade of repeated clinical errors, unnecessary medical interventions (such as the continued use of AFOs), and unwarranted cardiology follow-ups. Furthermore, this misdiagnosis carried severe psychological and physical consequences for the patient and his family. Living with the presumed risk of a sudden aortic dissection causes significant anxiety, situational depression, and psychosocial stigmatization, particularly regarding exercise restriction during childhood (16).

Since the advent of NGS, Medical geneticists have become a “key element” required to correctly interpret complex genetic reports and provide adequate counseling to families. Recently, experts have emphasized the necessity of building multidisciplinary healthcare teams with genomics expertise (44). Martin-Sanchez et al. proposed expanding these educational programs across six professional profiles, specifically identifying 58 core competencies required to achieve the full implementation of personalized precision medicine in clinical practice (45). Regrettably, in countries like Spain, the implementation of such programs is hampered by the lack of an officially recognized Clinical Genetics medical specialty. This institutional void limits the number of geneticists in hospitals and hinders the protocolization of genetic studies.

## 5 Conclusion

The interpretation of clinical genetic information and its communication to patients are by no means trivial tasks. As illustrated by this case, errors in this process generate severe physical, psychological, and financial burdens for patients and healthcare systems alike. As we move toward the ideal scenario of personalized precision medicine—where multidisciplinary teams include clinical professionals, geneticists, data scientists, and bioinformaticians (46)—it is imperative that all suspected genetic cases be promptly referred to specialized genetics units. Only through collaborative, multidisciplinary evaluation, supported by rigorous functional validation of genomic variants, can we prevent cascading errors and successfully shorten the diagnostic odyssey for patients with rare diseases.

## Data Availability

The datasets presented in this study can be found in online repositories. The names of the repository/repositories and accession number(s) can be found in the article/Supplementary Material.
