# Supplementary material for: Case Report: Diagnostic odyssey in rare diseases: when genetic variants are misinterpreted
Source: Front Pediatr. 2026 Apr 15;14:1803924. doi: 10.3389/fped.2026.1803924 (PMC13127307; doi:10.3389/fped.2026.1803924)
Supplement: Supplementary file 1 [file Datasheet1.pdf]

Suplementary Table 1. Score of systemic characteristics of Marfan Syndrome.

Systemic findings	Score
Wrist sign and thumb sign	3
Wrist sign or thumb sign	1
Pectus carinatum	2
Pectus excavatum or thoracic asymmetry	1
Hindfoot deformity	2
Flat feet	1
Acetabular protrusion	2
Upper body segment/lower body segment ratio reduced and arm/height ratio increased.	1
Scoliosis or thoracolumbar kyphosis	1
Reduced elbow extension	1
Pneumothorax	2
Dural ectasia	2
Facial features (dolichocephaly, enophthalmos, low palpebral fissures, malar hypoplasia, retrognathia) (3/5)	1
Skin striae	1
Myopia (>3 diopters)	1
Mitral valve prolapse	1

<u>Targeted Gene Panel</u>

Exome sequencing of a targeted gene panel of 10 genes (*ACTA 2* (HGNC:130), *COL3A1* (HGNC:2201), *COL5A1* (HGNC:2209), *COL5A2* (HGNC:2210), *FBN1* (HGNC:3603), *FBN2* (HGNC:3604), *MYH11* (HGNC:7569), *SMAD3* (HGNC:6769), *TGFBR1* (HGNC:11772) and *TGBR2* (HGNC:11773)) associated to MFS and related disorders was carried out in the proband by an external company. Approximately, 99.20% of exonic regions and splice-site junctions were reliably sequenced with, at least, 20× coverage.

Capture and enrichment of the exonic and intronic flanking regions of the genes contained in the MedExon (Roche) sequencing panel was performed with Roche NimbleGen SeqCap EZ HyperCap Lybrary^{TM} technology. NGS was performed with the NextSeq^{TM} sequencer (Illumina). Sequencing data were aligned to the reference genome (build 37 of Hg19 genome) and variants were further annotated and analyzed using Alamut visual^{TM} (Interactive Biosoftware), Ingenuity variant analysis^{TM} software (Quiagen), Variant interpreter^{TM} (Illumina) and VarAFT^{TM} software. The following variant databases were used: dbSNP, 1000 Genomes and gnomAD, Human Gene Mutation Database (HGMD; 2019.4 version), ClinVar and LOVD.
